# P012: Why a new definition for central line–associated bloodstream infection is necessary for surveillance in immunocompromised patients

**DOI:** 10.1186/2047-2994-2-S1-P12

**Published:** 2013-06-20

**Authors:** RE Quirós, A Novau, L Fabbro, M Casanova, G Kremer, M Pereyra

**Affiliations:** 1 Prevention and Infection Control Department, Hospital Universitario Austral, Caba, Argentina

## Introduction

Accurate surveillance definitions are necessary to evaluate the impact of interventions to prevent central line–associated bloodstream infections (CLABSIs). Although, the National Healthcare Safety Network (NHSN) definition for CLABSI has been applied extensively in intensive care units, few studies have examined its performance among bone marrow transplant (BMT) recipients. As those patients have inherent risks for bloodstream infections associated with mucosal barrier injury, more specific definitions are necessary for catheter-related bloodstream infection (CRBSI) in order to determine the impact of improvement projects to decrease these device-associated infections (DAIs).

## Objectives

To determine the impact of a care bundle in the CRBSI rates in a BMT population using a specific definition.

## Methods

Since Feb’10, a care bundle for insertion and maintenance of central venous catheter was implemented across our institution. While CLABSI was defined using NHSN criteria, a definitive diagnosis of CRBSI required that the same organism grew from at least 1 percutaneous blood culture and from a semiquantitative culture of the catheter tip, or that 2 blood samples drawn, one from a catheter hub and the other from a peripheral vein, showed differential time to positivity (DTP) ≥ 2 hours. Episodes with a DTP < 2 hours were categorized as catheter-non related bloodstream infection (CNRBSI). The impact of care bundle was estimated comparing 2012 vs 2009, CRBSI and CNRBSI incidence density rates.

## Results

While the CRBSI rate showed a reduction from 8.94 per 1,000 central line–days to 2.93 per 1,000 central line–days (difference 6.01; CI 95%>1.59 to 13.61, p=0.07), CNRBSI showed a marginal reduction from 5.96 per 1,000 central line–days to 4.69 per 1,000 central line–days (difference 1.28; CI 95%>5.41 to 7.96, p=0.68). While in CNRBSI intestinal organisms predominated (79%, 23/29), the most common organisms isolated in CRBSI was coagulase-negative *Staphylococcus* (78%, 14/18).

## Conclusion

Using DTP as part of CRBSI definition allowed us to accurately assess the impact of a care bundle in the reduction of these DAIs.

## Disclosure of interest

None declared

